# Rest, replace, and recover: TandemHeart to transcatheter aortic valve replacement—a case report

**DOI:** 10.1093/ehjcr/ytae465

**Published:** 2024-09-02

**Authors:** Syed H Haq, Sidra R Shah, David Eapen, Anna Kleman, Mallory Knous, Amanda Laird, William Cole, Sandeep M Patel

**Affiliations:** Department of Internal Medicine, Bon Secours Mercy Health–St. Rita's Medical Center, 730 W Market Street, Lima, OH 45801, USA; Department of Internal Medicine, Bon Secours Mercy Health–St. Rita's Medical Center, 730 W Market Street, Lima, OH 45801, USA; Department of Internal Medicine, Bon Secours Mercy Health–St. Rita's Medical Center, 730 W Market Street, Lima, OH 45801, USA; Structural Heart & Intervention Center, Bon Secours Mercy Health–St. Rita's Medical Center, 730 W Market Street, Lima, OH 45801, USA; Department of Critical Care Medicine, Bon Secours Mercy Health–St. Rita's Medical Center, 730 W Market Street, Lima, OH 45801, USA; Department of Critical Care Medicine, Bon Secours Mercy Health–St. Rita's Medical Center, 730 W Market Street, Lima, OH 45801, USA; Department of Critical Care Medicine, Bon Secours Mercy Health–St. Rita's Medical Center, 730 W Market Street, Lima, OH 45801, USA; Structural Heart & Intervention Center, Bon Secours Mercy Health–St. Rita's Medical Center, 730 W Market Street, Lima, OH 45801, USA

**Keywords:** Cardiogenic Shock, TandemHeart®, Transcatheter aortic valve replacement, Case report

## Abstract

**Background:**

Severe aortic stenosis (AS) can present insidiously, with the end stages resulting in significant valvular–induced cardiomyopathy and can lead to cardiogenic shock (CS). Such cases result in a myriad of complex manifestations and are often associated with a poor prognosis. These patients require emergent cardiac evaluation and valvular intervention. Unfortunately, the immediate nature of the CS provides little time for a detailed valvular evaluation. Possible management involves use of mechanical circulatory support (MCS) prior to urgent transcatheter aortic valve replacement (TAVR).

**Case summary:**

The patient was a 70-year-old female who developed refractory CS, and acute decompensated heart failure was complicated by AV block secondary to severe AS. Due to progressively worsening haemodynamics, the need for MCS for cardiovascular support and eventual valve replacement resulted in the decision to pursue TandemHeart® (TH; LivaNova Inc, Pittsburgh, PA, USA). We discuss the novel implementation of the TH as a means of bridging to TAVR.

**Discussion:**

TandemHeart system provides the benefits of improving haemodynamic support in CS while allowing unencumbered access to the stenotic valve for balloon aortic valvuloplasty (BAV) or TAVR. In our evaluation, we discuss the utilization and benefits associated with TH to TAVR in allowing for cardiac rest, replacement of the valve, and recovery of left ventricular function.

**Learning points**

- To understand the use of mechanical circulatory support for cardiac recovery and bridge therapy
- To compare the benefits and differences of TandemHeart over other mechanical circulatory support with TAVR

## Introduction

The severity and associated symptoms of aortic stenosis (AS) are contingent on numerous variables and subject to the degree of valve degeneration along with other coexisting cardiac pathologies including ischaemic heart disease.^1^ No pharmacologic therapy has been proven to show deceleration of disease progression. Given that severe AS is associated with a high mortality rate, up to 50% if left untreated, time is an unaffordable luxury.^2^ As such, it is not uncommon for the patient to also develop CS, propelling their cardiovascular failure and further exacerbating the complexity of management. Temporizing measures with balloon aortic valvuloplasty (BAV) need to be done cautiously, often in conjunction with inotropic and vasopressor support to ensure haemodynamic stability. Regrettably, this prolongs cardiac recovery, risks arrhythmogenic states, and increases overall mortality. An alternative mode of achieving haemodynamic stability and allowing for cardiac rest is the utilization of mechanical circulatory support (MCS).^3^ The use of MCS to bridge for transcatheter aortic valve replacement (TAVR) in critically ill and unstable situations has been discussed before.^4^ However, the discussion of TandemHeart (TH) use as a conduit to definitive therapy with TAVR remains elusive to current literature. We present a unique case of AS-induced CS stabilized with TH allowing for definitive therapy with TAVR and discuss the interventional approach and implications of this unique management strategy.

## Summary figure

**Figure:**
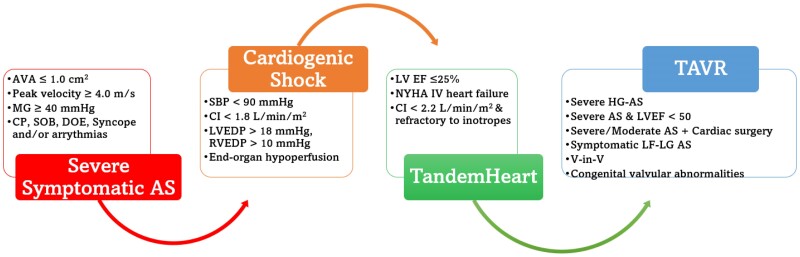


## Case presentation

A 70-year-old female with a past medical history of obstructive coronary artery disease (CAD) with prior coronary artery bypass × 3, hypertension, hyperlipidaemia, stroke, diabetes, chronic obstructive pulmonary disease, interstitial lung disease, chronic kidney disease, rheumatoid arthritis, and ulcerative colitis presented for non–ST segment elevation myocardial infarction with refractory bradycardia and hypotension. Telemetry showed intermittent 2:1 aortic valve (AV) block and runs of asystole. She remained refractory to atropine and vasopressors and progressively worsened. She subsequently was intubated and taken for an emergency catheterization. A transvenous pacer was placed, and patent grafts with moderate disease in the posterior descending artery were appreciated. During the procedure, her haemodynamics continued to worsen despite multiple pressor supports. A transoesophageal echocardiogram (TEE) demonstrated acutely dropped ejection fraction (EF) to 25%–30% from previous EF of 55%. Additionally, haemodynamic assessment demonstrated an aortic valve area of 0.9 cm^2^ and mean gradient of 27 mmHg. Cardiac index (CI) was 1.7 mL/min/m^2^, with a pulmonary artery saturation of 43%. It was evident the patient was in CS secondary to acute decompensated heart failure in the setting of severe AS. The patient was stated on inotropic support with little relief. Therefore, the decision for MCS placement was made to provide cardiac support until the patient was deemed stable for valve replacement.

**Figure 1:**
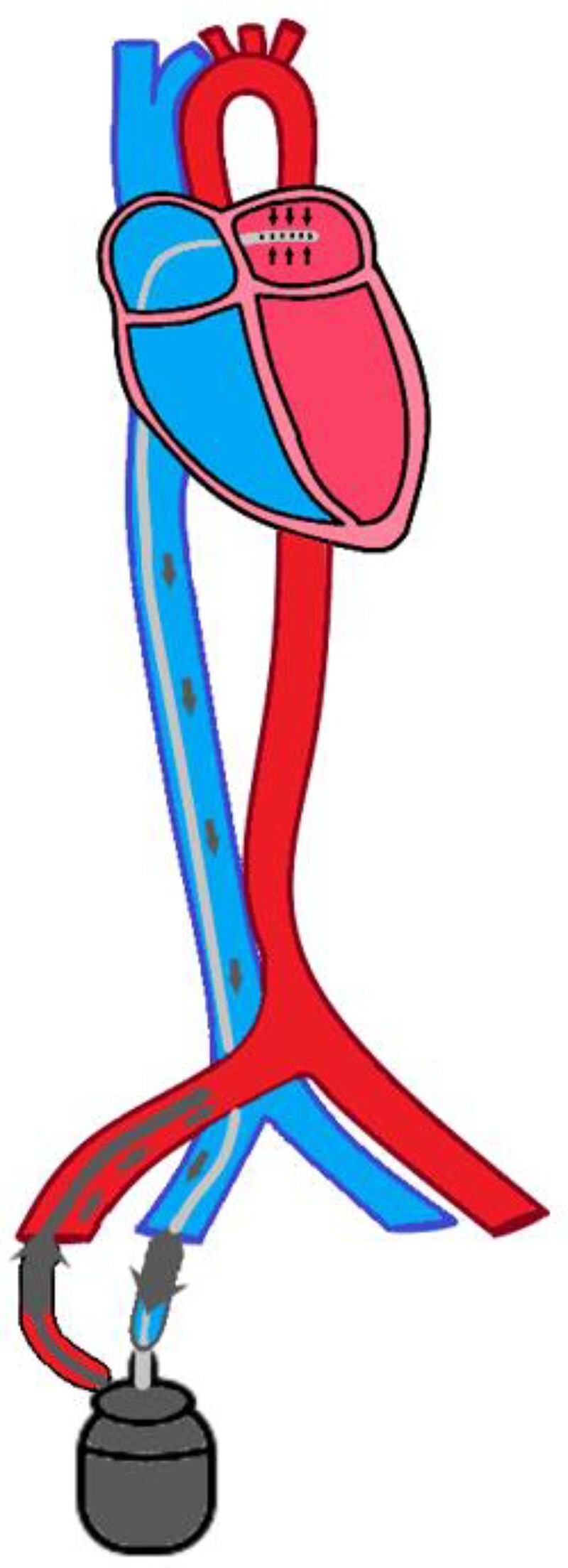
TH circuit. It involves a trans-septal puncture via a 21 Fr right venous cannula that provides venous drainage from the left atrium. This blood via a centrifugal is pumped back into the body via a 15–19 Fr right arterial cannula. Cardiac flow is maintained ranging from 2.5 to 5 L/min.

Initial considerations of Impella® device were deferred due to the eventual need for TAVR, thereby necessitating access to the aortic valve. Given this and the need for extensive cardiovascular support, the decision was made to proceed with TH. The system encompassed a 19 Fr right femoral artery access with a 6 Fr antegrade bypass and 21 Fr right femoral venous access through which trans-septal cannulation into the left atrium was completed. The TH circuit can be visualized in *Figure 1*. Following the procedure, the patient was transferred to the intensive care unit for close monitoring. Cardiothoracic surgery was consulted and deemed her not a surgical candidate for valve replacement due to her chronic and acute co-morbidities including lactic acidosis and acute renal and liver dysfunction. Instead, percutaneous intervention was opted for.

After 2 days on TH, she underwent TAVR with a 26 mm Evolut FX (EFX) (seen in Supplementary material online, *Video S1*). Post procedure, she was effectively weaned off inotropes and vasopressors, while maintaining a CI > 2.2 mL/min/m^2^ and a mean arterial pressure > 65 mmHg. TandemHeart was explanted 3 days later with intra-arterial balloon pump (IABP) inserted to assist with diuresis. Intra-arterial balloon pump was explanted another 3 days later, and the patient was extubated. A repeat transthoracic echocardiogram (TTE) showed EF 40%–45%, AVA 1.85 cm^2^, MG 4 mmHg, and velocity 2.6 cm/s with no residual AS or insufficiency. The patient ultimately recovered and was discharged to inpatient rehabilitation. She would go on to make a full recovery, released from rehabilitation and was evaluated 3 months later at outpatient cardiology clinic.

## Discussion

To the dismay of clinicians, end-stage severe AS often presents in a complex manner. Cardiogenic shock is very prevalent in these cases and sometimes with concomitant acute coronary syndrome or arrhythmias. The presence of CS makes treating AS extremely challenging and is associated with subpar outcomes, including a high mortality rate.^5^ Chemical temporizing measures are the mainstay with inotropy and vasopressor support until cardiac function has recovered well enough to tolerate definitive therapy without the risk of post-procedure decompensation. However, this measure can be time-consuming, thereby resulting in progressive organ deterioration, and further delaying valve replacement.

Aortic stenosis is a mechanical problem in which medical therapy is temporizing at best and ultimately results in continued organ failure and potentially death.^6,7^ Traditionally, severe AS was temporized and, in some cases, solely treated with BAV as it was seen as the only method of stabilization for AS and CS. Typically requiring a smaller bore arterial access and an undersized aortic balloon, BAV provides an immediate reduction in the gradient across the valve with improvement in haemodynamics. This allows the heart team time to decide the best course of action. The negative aspects of BAV include embolic CVA, arterial access site bleeding, restenosis/recoil of the aortic valve, and aortic insufficiency. Furthermore, the effect of BAV is usually temporary with effects lasting 1–3 months, thereby predisposing to the recurrence of CHF and CS.

Transcatheter aortic valve replacement provides the most definitive course of therapy since it rids the patient of the initial inciting cardiac insult. It has gained traction as a relatively available and less invasive alternative to surgical AVR (SAVR). Patients who received TAVR vs. SAVR in CS had lower mortality rates, 19% vs. 26%, respectively.^5^ This is especially true as the incidence of adverse vascular complications has decreased, owing in part to operator techniques, advanced valve and apparatus design, reduced sheath size, and improved closure devices.^2,8^ Despite this, limitations include concomitant CAD management, pre-procedural stroke assessment, and prevention, and valve durability are areas of concern for a lower risk and younger population.^2^ Although TAVR has proven to be a viable treatment course in acute cardiogenic shock, there remains an elevated risk of death at 30 days and up to 1 year post procedure, this typically contingent on the timing and degree of CS.^9^

The use of MCS prior to urgent TAVR may serve to mitigate the detrimental course of CS. MCS functions by supplementing the heart during CS or while bridging to definitive therapy, such as with complex PCIs. The most common cardiac support device, Impella CP, is widely utilized in CS (*Table 1*). The Protect II and IMPRESS trial compared IABP to Impella® and found the latter to provide greater cardiac power output, although there was no difference in 30-day mortality.^4,10^ However, the notion of Impella’s superiority among other MCS has been challenged with a recent retrospective study showing increased mortality within the Impella® groups when compared to TH in CS.^11^ This was re-demonstrated in a nationwide analysis comparing both devices in CS during which Impella® was associated with a higher mortality (*P* < 0.0001), although the reported hospitalization stay and associated costs were lower (*P* < 0.001).^12^ Although TH possess relatively less cost, this advantage may be offset by an overall lengthier hospitalization stay associated with TH when compared to Impella®. It is important to note this is contingent on institutional standards and expertise. Finally, in cases with severe AS and CS, the trans-valvular location of the pump inherently prevents simultaneous interventions on the aortic valve while maintaining haemodynamic support. These drawbacks are reflected in a recent study on TAVR outcomes in patients with CS that demonstrated an increased mortality with MCS, particularly the Impella® device.^13^ Alternatively, the TH system is a strictly left ventricular bypass option utilizing a trans-septal left atrial cannula connected to a motorized heart pump that directs oxygenated blood back into the aorta via femoral arterial access. In doing so, it augments flow and alleviates the burden on the left heart. Randomized trials with TH in CS have shown improved CI, mean arterial pressure, and decreased pulmonary capillary wedge pressures leading to improved survival outcomes.^11^ This was further illustrated in the THEME registry, a multicentre prospective observational study that analysed haemodynamic changes and outcomes with TH in CS. It found that TH utilization demonstrated a 74% 30-day and 66% 180-day survival benefit.^14^

**Table 1:** Various benefits and limitations of contemporary mechanical circulatory support used to provide left ventricular support in a state of cardiac failure or shock

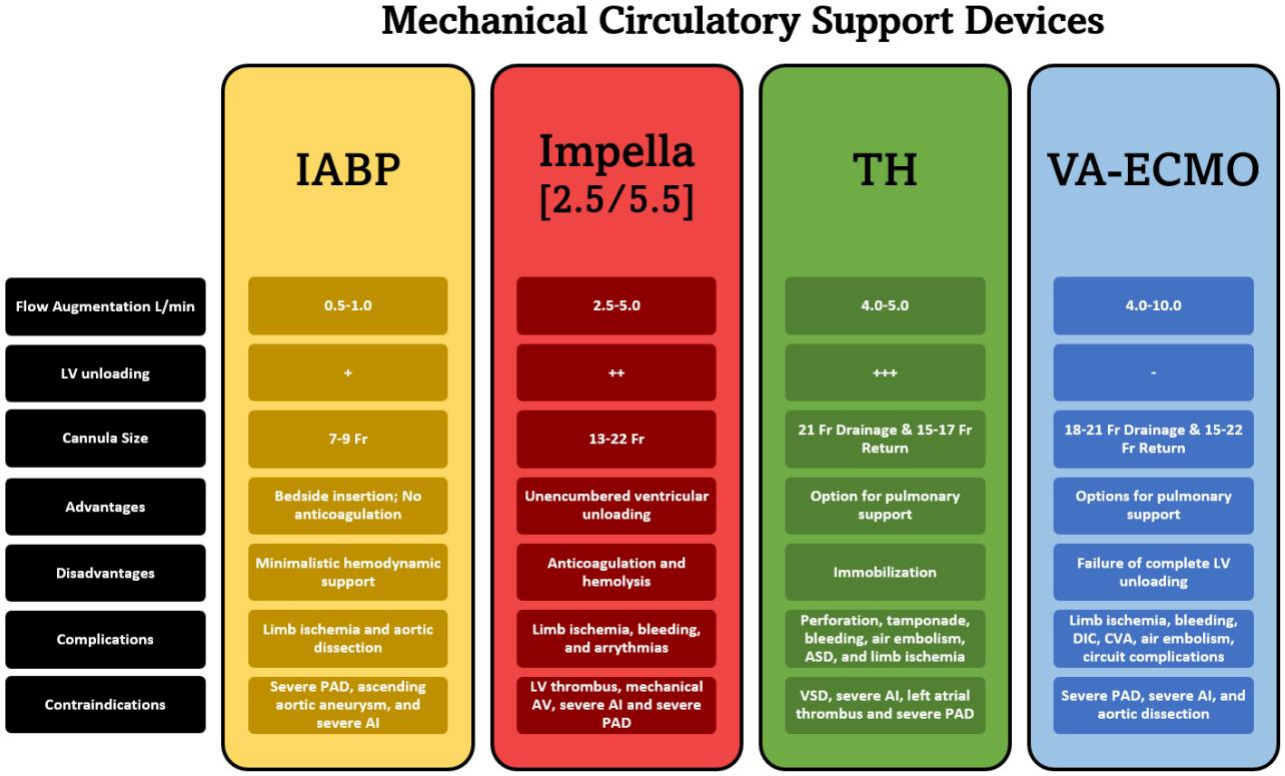

**Figure 2:**
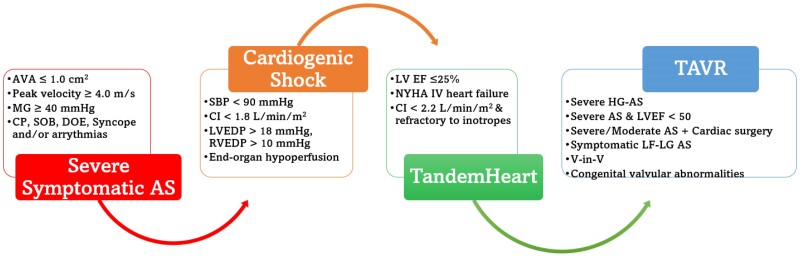
Generalized schematic approach to TandemHeart to transcatheter aortic valve replacement bridge therapy.

In severe AS with concomitant or impending CS, clear foresight of treatment methodology is necessary to circumvent a rapidly deteriorating state. Once TAVR is decided for definitive therapy, computed tomographic imaging is paramount to a successful procedural outcome via optimal valve selection and deployment. During this process, MCS can provide the left ventricular myocardium rest while allowing the heart team time to prepare the appropriate replacement valve. Among MCS options, the Impella® device may alter TAVR access site and result in significant artefact when performing valvular/vascular measurements which can affect valve sizing and planning. The Impella® lies in the aorta and is situated across the aortic valve. Depending on the patient’s physiology or physical limitations, this can pose the risk of entanglement or obstruction during the valve deployment process as the prosthesis is advanced with the Impella® in place up to the aortic valve. Further, the Impella® device must be removed while placing the TAVR, implying a period without support during valvular deployment. Contrary to this, the TH circuitry allows the patient to remain fully supported despite any haemodynamic perturbations that may occur while performing TAVR. Despite TAVR placement, most patients continue to require further MCS post deployment to allow for complete recovery—in the setting of Impella® that means placing an entirely new Impella CP catheter across the new prosthesis, a process that can predispose the prosthesis leaflet to unintentional damage. In fact, this necessitated a Class 1 FDA recall, followed by a review and update of the instructions for Impella® use. Alternatively, the TH can remain in position without adjustments of circuitry pre-, intra-, and post-TAVR, with adjustments of pump flow dictated by the patient’s clinical status. Finally, in general, the TH provides greater overall cardiovascular support than its counterpart, the Impella CP (*Table 1*), which ultimately enables both tailoring of flow and early de-escalation of vasopressors/inotropes. For these reasons, TH becomes the clear choice when deciding on MCS while contemplating TAVR (*Figure 2*). However, cons associated with this approach include large bore access, need for systemic anticoagulation, performance of acute trans-septal puncture, aggressive volume loading, high rate of transfusions, haemolysis, and acute limb ischaemia; these risks need to be tempered with the overall benefit before deciding on this approach and may be reduced by early valvular replacement to avoid the deleterious effects of the TH system which occur with prolonged use.

## Conclusion

Severe AS can follow a rapidly decompensating course if not intervened upon in a timely fashion. This is further complicated and propagated by CS, making treatment extremely difficult and challenging. In such patients, mortality rates are high as time to TAVR is delayed in the setting of acute decompensated heart failure. In these situations, TH can be an attractive method to allow for resting of the left ventricle, replacement of the aortic valve, and recovery of the overall cardiac function. Larger trials are warranted to further investigate the impact of this thesis.

## Supplementary Material

ytae465_Supplementary_Data

## Data Availability

There is no new data generated in this paper in support of the subject matter.

## Lead author biography

Dr. Syed Haq is a resident physician at Mercy Health, St. Rita’s Medical Center in Ohio, USA. He graduated from the medical school at Burrell College of Osteopathic Medicine in 2021. He has interests in interventional cardiology and critical care.
